# The Greatest Learning Return on Your Pedagogical Investment: Alignment, Assessment or In-Class Instruction?

**DOI:** 10.1371/journal.pone.0137446

**Published:** 2015-09-04

**Authors:** Emily A. Holt, Craig Young, Jared Keetch, Skylar Larsen, Brayden Mollner

**Affiliations:** Department of Biology, Utah Valley University, Orem, Utah, United States of America; Oregon State University, UNITED STATES

## Abstract

Critical thinking is often considered an essential learning outcome of institutions in higher education. Previous work has proposed three pedagogical strategies to address this goal: more active, student-centered in-class instruction, assessments which contain higher-order cognitive questions, and greater alignment within a classroom (i.e., high agreement of the cognitive level of learning objectives, assessments, and in-class instruction). Our goals were to determine which of these factors, individually or the interactions therein, contributed most to improvements in university students’ critical thinking. We assessed students’ higher-order cognitive skills in introductory non-majors biology courses the first and last week of instruction. For each of the fifteen sections observed, we also measured the cognitive level of assessments and learning objectives, evaluated the learner-centeredness of each classroom, and calculated an alignment score for each class. The best model to explain improvements in students’ high-order cognitive skills contained the measure of learner-centeredness of the class and pre-quiz scores as a covariate. The cognitive level of assessments, learning objectives, nor alignment explained improvements in students’ critical thinking. In accordance with much of the current literature, our findings support that more student-centered classes had greater improvements in student learning. However, more research is needed to clarify the role of assessment and alignment in student learning.

## Introduction

Critical thinking is an imperative skill set, often regarded as one of the top hiring criteria by employers today [1]. Ennis [2] proposed that critical thinking involves several abilities related to clarity, inference, establishing a basis for inference, and decision-making. In theory, the successful achievement of these skills enables one to participate and contribute as a learned citizen in our society [3–5]. However in the context of higher education, critical thinking is often more narrowly defined as simply higher-order cognitive skills. Halpern [6] defines higher-order cognitive skills as “skills that are relatively complex; require judgement, analysis, and synthesis.” In addition, science education practitioners and researchers often include more complex application as a higher-order skill [7–9]. These higher-order cognitive skills contrast with lower-order cognitive skills which are restricted to simple tasks (e.g., recollection, recognition, clarification, classification, explanation, or simple application) [7, 10].

Increased pressure is placed upon institutions of higher education to produce graduates who demonstrate proficiency with these higher-order cognitive skills. Effectively teaching students to develop competency in critical thinking and translate these skills into expertise in the work force has been a subject of much research. Three aspects of teaching have been the focus for development of these skills in the university classroom: in-class instruction, assessment, and course alignment.

In-class instruction, and its myriad of pedagogies, has been extensively researched to identify its role in learning. Freeman et al. [11] unequivocally demonstrated that learner-centered pedagogies are associated with greater learning gains and lower failure rates compared to teacher-centered approaches in science, technology, engineering, and math classes. Moreover, traditional, didactic lectures (i.e., teacher-centered teaching) promote mastery of lower-order cognitive skills, such as memorization and recall [3]; while, learner-centered teaching (e.g., constructivism, active learning, collaborative learning) can have a positive impact on improving both students’ lower and higher-order cognitive skills [12–18]. Engaged teaching strategies can improve these skills through opportunities for students to practice constructing and evaluating knowledge [19], and scaffolding such skills through support and modeling by student peers and instructors [20].

Second, assessment can both measure and promote higher-order cognitive skills. Summative assessments can verify students’ understanding of the material they learn and provide objective criteria to assign grades; while, formative assessments can gauge the effectiveness of teaching and contribute to a student’s metacognition [21–23]. Theoretically, practice and feedback provided by formative assessments manifest in higher summative assessment scores [24]. By example, Barak and Dori [25] designed assessments in a graduate course for science teachers to incorporate higher-order cognitive skills and subsequently improved their students’ overall critical thinking. However, only Jensen et al. [26] have directly compared how different assessments, reflecting a continuum from lower to higher-order skills, affect students’ overall critical thinking.

Bloom’s taxonomy of learning [9–10, 27] has been an important and helpful tool to conceptualize differences among types of cognitive tasks often found in assessments and learning objectives. Regretfully, reports indicate that assessment items in STEM courses tend to focus on lower-order cognitive skills (e.g., knowledge, comprehension, and less frequently application) and rarely, if ever, test higher-order cognitive skills [28–29]. Current research indicates that the prevalence of lower Bloom level assessments can hamper students’ critical thinking, while assessments at higher Bloom levels can increase students’ critical thinking [26].

Further, in theory, assessments are intricately tied to learning objectives. Through backward design faculty are encouraged to formulate educational goals (i.e., learning objectives) before assessments are designed [30]. Faculty expectations of students can be set early in a course by using learning objectives to outline what students must be able to do and know after instruction [29, 31]. Learning objectives can assist faculty in developing class activities and assessments which match the same content and cognitive levels, and these objectives provide criteria on which a student is evaluated [21, 31]. Learning objectives can also guide students in preparing for summative assessments [28]. The condition in which criteria match among learning objectives, classroom instruction, and assessments is known broadly as alignment [21, 32].

In post-secondary education, many types of alignment exist [21, 32]. These different types of alignment may be focused on separate or combined aspects of a classroom or program such as curriculum, learning standards, assessments, or policy. In this paper, we use a narrow definition of alignment, focused within a single classroom. Our alignment is the agreement between the Bloom’s taxonomy level of an instructor’s learning objectives and the Bloom’s level of their assessments. While many definitions also include an in-class pedagogy element, our measure of learner-centeredness reflects more than just the cognitive level of learning but also quantifies classroom culture. Therefore we focused our investigation of alignment on the correspondence of the cognitive level of learning objectives and assessments, and did not integrate in-class practices in our definition.

Aligning a classroom helps form a “coherent model of curriculum, pedagogy, and assessment” [33]. This coherence is perceived as beneficial, because alignment can help instructors develop course materials and ensure their students reach the desired educational goals [33]. Specific tools such as curriculum mapping are used to help instructors and departments evaluate the extent to which courses are in alignment with learning objectives [34, 35].

Alignment is often associated with “good teaching” [31], and has been predicted to increase student learning [36–40]. Unfortunately, misalignment of learning objectives, in-class instruction, and assessments is commonplace [28, 34, 41–43]. Moreover, no previous study has provided a quantitative link between greater alignment, measured by Bloom’s taxonomy level, and increases in student learning [38], or its relative weight compared to other pedagogical practices. While higher-order cognitive skills achievement has been linked individually to in-class instruction techniques [18, 44–47], the level of assessments [48], and classroom alignment [21, 38, 49–51], no single study has examined their relative and interactive effects on student learning gains. Our study investigates the combination of all three instructional strategies in a novel way to ask what drives student improvement on higher-order cognitive skills.

Our two research questions were: (1) Would students in General Biology courses, on average, perform better on post quizzes that assessed their biology-content higher-order cognitive skills compared to pre quizzes administered earlier in the same semester? (2) Which pedagogical elements (i.e., the cognitive level of assessment, the cognitive level of learning objectives, classroom alignment, the learner-centeredness of the classroom, or any interaction therein) contribute to improvements in students’ higher-order cognitive skills?

## Materials and Methods

### Ethics Statement

The Institutional Review Board of Utah Valley University approved the procedures for the study (IRB# 01103). Written informed consent was obtained by all participating students and faculty at the beginning of the study.

### Participants and survey instrument

We chose to conduct an observational study of several un-manipulated introductory biology classrooms to investigate our two research questions. We believe that the classrooms we observed represent the norm of biology classrooms drawn from a public post-secondary institution in the western US. As a primarily undergraduate institution, an emphasis on teaching and learner-centered approaches is encouraged institution-wide; however, none of the participating faculty have “exceptional teaching expertise” [52] deeming their pedagogies as outlying. We hoped that this approach would allow us to draw conclusions to an average biology classroom.

We sampled 1114 students from fifteen sections of a general biology course for non-majors. These fifteen class sections were taught by one of nine participating instructors over two semesters, Fall 2013 and Spring 2014. In our fifteen participating class sections, student enrollment ranged between 391 and 16 students (mean = 89.4 students per section). One of these sections was a weekend course, 20% were night classes, and the remaining eleven sections were taught during the weekday. We administered two voluntary quizzes each semester, one the first week and one the last week of each semester. These quizzes were available to students online through Survey Monkey, www.surveymonkey.com. While independent of each of the individual courses’ assessments, completion of these quizzes was worth 2% of each students’ final grade.

Each pre and post-quiz consisted of two parts, a demographic questionnaire and a biology-content cognitive skills quiz. The demographic portion, including seven questions, collected the ethnic and educational backgrounds of the student participants. The cognitive skills quiz consisted of twelve questions that covered a range of biology topics that addressed the essential learning outcomes determined by the local biology department and some of the core concepts and competencies of Vision and Change [53], a report calling for change in undergraduate biology education by the American Association for the Advancement of Science and National Science Foundation (Table 1). While multiple factors of the departmental learning outcomes and Vision and Change [53] core concepts and competencies are represented in our quiz (Table 1), we only used the summed score from all items, thus we did not explore the internal factor structure of any redundant items.

**Table 1:** Summary of higher-order questions used on the pre- and post-quizzes.

Pre-question	Bloom’s level	Post-question	Bloom’s level	Department essential learning outcome	Vision & Change Core Concepts & Competencies	Source
Radish	5	Maple tree	3	Communication	Pathways and transformation of energy and matter, Systems	Ebert-May et al. 2003
Jared	3	Penguin	3	Communication	Pathways and transformation of energy and matter, Systems	Wilson et al. 2006
Chromatids	3	Chromatids^a^	3	Communication	Structure and function	Shi et al. 2010
Codon, mRNA	4	Codon, mRNA	4	Communication	Information flow, exchange, and storage	Smith et al. 2008
Codon, protein	4	Codon, protein	4	Communication	Information flow, exchange, and storage	Smith et al. 2008
Phospholipid	3	Phospholipid	3	Quantitative reasoning	Structure and function	Shi et al. 2010
Ginseng	3	Iron	3	Critical, analytical, and creative thinking	Systems; Ability to apply the process of science, ability to understand the relationship between science and society	Sirum & Humburg 2011
Cheetah^a^	5	Salamanders^a^	5	Information literacy	Evolution	Nehm & Reilly 2007
Climate change^a^	2	Climate change^a^	2	Information literacy	Systems	Lombardi & Sinatra 2012
Hexokinase	3	Hexokinase	3	Technical literacy	Structure and function, Information flow, exchange and storage	Shi et al. 2010
Pedigree	4	Pedigree	4	Technical literacy	Structure and function, Information flow, exchange and storage; Ability to use modeling and simulation	Smith et al. 2008
Spider Monkey	5	Grandma Johnson	5	Local community	Pathways and transformation of energy and matter, Systems	Ebert-May et al. 2003

^a^Three questions excluded from the analyses.

The cognitive skills portion of our survey instrument was assembled to balance the maximum number of introductory biology topics, to ensure all participating instructors taught most of the content, with the shortest length of quiz to achieve the greatest student response rate. Quiz questions were compiled from all or portions of published and validated concept inventories [48, 54–59]. Moreover, we aimed to select questions that evaluated higher-order cognitive skills, represented by a Bloom’s Taxonomy of Learning level of three (i.e., application) or above.

Three of the quiz questions in the cognitive skills portion were free response and the other nine were forced-response multiple choice questions (Table 1). We evaluated students’ free responses, by question, using published rubrics for each question [54, 56, 58]. Ten raters were trained by the first author (EH) to use the three rubrics. Based on a subset of student responses, the two most consistent raters for a given free-response question rated the remaining responses of that question (both pre and post-quiz). The first author conducted a blind audit of 20% of all free-response ratings, by question. Using Cohen’s weighted Kappa [60] to scale the magnitude of disagreement using linear and equal weights, we found high inter-rater reliability (K_w_: ginseng/iron = 0.76, cheetah/salamander = 0.77, spider monkey/Grandma Johnson = 0.93).

The proportion of correct responses on the cognitive skills quiz taken at the end of the semester represents the response variable throughout our study. All questions were weighted equally in this calculation. Multiple choice questions were scored as either correct or incorrect. Rubrics for the open-response questions allowed for partial credit on these three questions. Given the relatively poor student performance on the individual concept inventory which contributed to our biology-content cognitive skills quiz [52, 56–59], we did not expect a normal distribution of scores (i.e., with a mean centered near 75%). Further since this was an observational study, we did not attempt to align the survey instrument with instruction in the participating sections. In sum, our quiz was intended to serve as a fine-toothed measure of higher-order cognitive skills. The practical significance of a one-point improvement on our quiz does not translate into a similar change on a quiz that may have been part of the class, because small improvements we measure likely signify large gains in critical thinking development.

### Bloom and alignment

We collected learning materials from each of the nine instructors, including syllabi, learning objectives, and assessments. The six instructors who taught two sections in the study, verified that these materials were identical for both of their two sections. Learning objectives (LOs) describe the cognitive skills and concepts the students were expected to learn. We collected 418 total LOs from all instructors, averaging 46.6 LOs per instructor; the greatest number of LOs for one instructor equaled 119, while another instructor listed only three. The structure and emphasis of LOs varied among instructors. Some described overarching themes for the entire class, often included as part of the syllabus, while others were narrowly defined objectives specific to an assessment or topic.

Of our nine participating instructors, almost half of them gave four high-stakes assessments throughout the semester. Three of our instructors gave five high-stakes assessments, one gave nine assessments, and one gave twenty quizzes throughout the term. We evaluated 2656 total assessment items (i.e., exam or quiz questions), which represented an average of 259.1 items per instructor (minimum = 71 items per instructor, maximum = 633 assessment items per instructor). These assessments represented a significant portion (at least 45%) of their students’ final grade. In addition to the assessments we evaluated, many of our participating instructors also reported administering lower-stake assessments (e.g., pop quizzes, clicker questions). Interviews with these faculty confirm that the cognitive level of these low-stakes assessments were similar to their high-stakes assessments. However we believe that most of these low-stakes assessments, despite their regular frequency throughout the semester, were also likely summative and not formative. According to Sadler’s [61] definition, assessments, where students are simply provided a grade or verification of a correct response without assistance on actions necessary to reach the standard, are summative. On average, our faculty reported that feedback was not individualized or only available in office hours; therefore, many or most of their students may have had limited access to necessary feedback.

We used Bloom’s Taxonomy of Learning to rate the cognitive level of learning of all learning objectives and assessment items on a scale from one to six (1 = knowledge, 2 = comprehension, 3 = application, 4 = analysis, 5 = synthesis, 6 = evaluation) [9–10, 29]. Seven raters were trained by the first author (EH) to determine Bloom levels using the Blooming Biology Tool [9] and descriptions in Anderson et al. [10]. LOs and assessments were divided non-randomly, and independently rated by two raters. Inter-rater reliability was adequate to good (K_w_: LOs = 0.53, assessments = 0.72). For each instructor, we calculated an arithmetic mean of all their LOs, hereafter referred to as the LO Bloom score (i.e., Bloom levels refer to items while Bloom scores refer to means of item levels). While we realize that a given instructor’s course likely has LOs that represent a range of Bloom levels, for our sample, the median and mean of these levels are virtually identical thus we opted for the mean to gain more precision in the estimate.

For assessments, we calculated the weighted average of Bloom levels for assessment items across all of an instructor’s assessments, using individual question point values as weights, according to Momsen et al. [29]. An instructor’s weighted assessment Bloom score represents the sum of the products of the Bloom level for each assessment item and the question weight, divided by the sum of the weights (or total points possible). We used raw Bloom scores of both assessments and LOs as continuous explanatory variables in regression analyses, and used the average mean value among all sections to create two categorical variables each with two levels of Blooms scores (i.e., high and low, with respect to the mean) used in ANCOVA analyses.

Using the Bloom scores from LOs and assessments, we calculated alignment scores for each instructor. An alignment score was the absolute value of the learning objective Bloom score minus the assessment Bloom score. An alignment score of zero represents perfect alignment, where students were assessed at the same cognitive level as they were expected to learn (e.g., if learning objectives, on average, expect students to *comprehend* biology content and then assessments in that course, on average, asked students to simply *comprehend* biology content). We chose to use mean Bloom scores in this calculation, rather than linking individual learning objectives with individual assessment items and averaging those differences, because in many cases assessment items could be linked to multiple learning objectives or a direct link was unclear.

**Fig 1:**
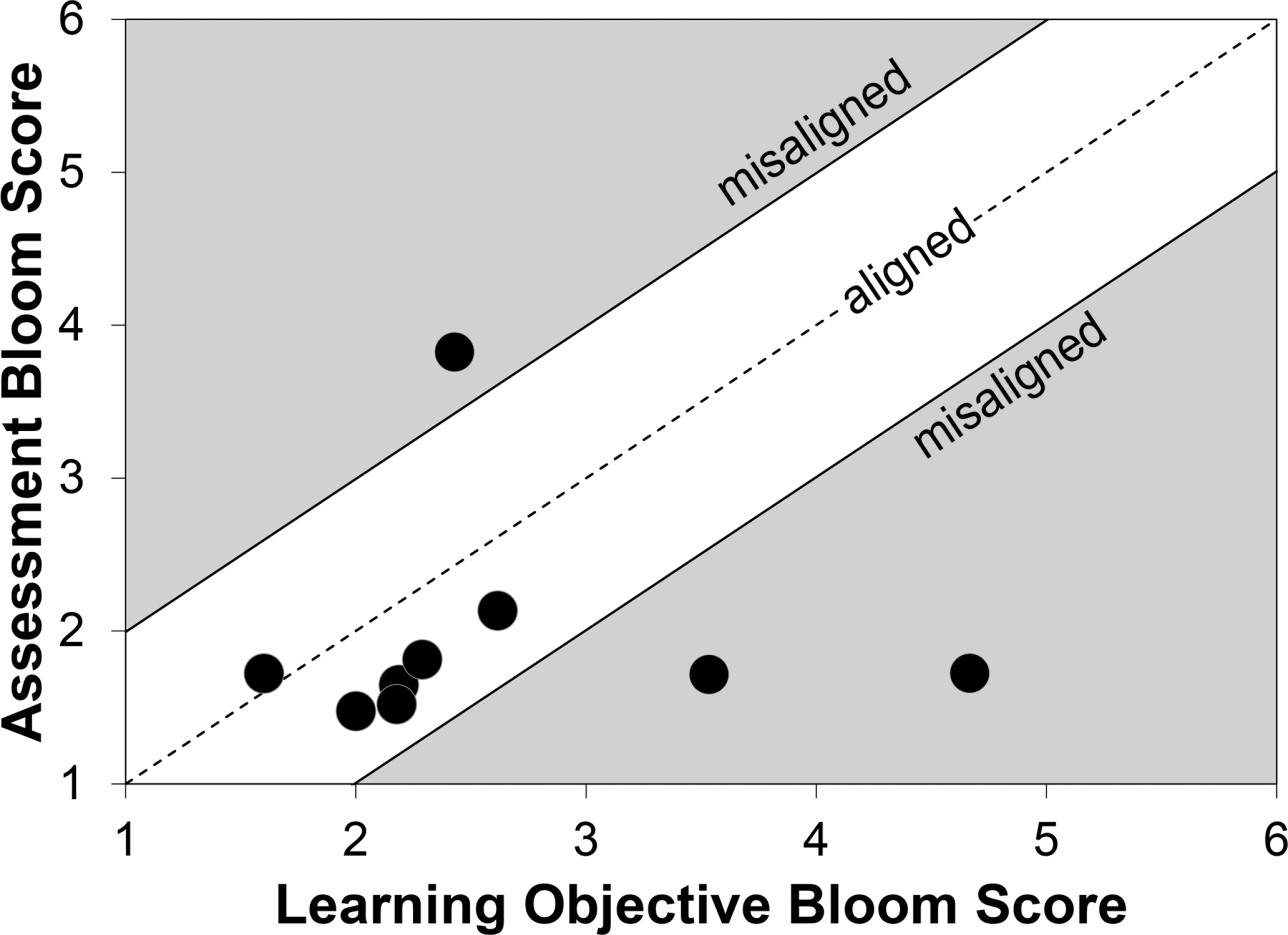
Scatterplot of learning objective and assessment Bloom scores. Bloom scores range from one to six (1 = knowledge, 2 = comprehension, 3 = application, 4 = analysis, 5 = synthesis, 6 = evaluation). The alignment score is the absolute value of the learning objective Bloom score minus the assessment Bloom score. Zero represents perfect alignment, while any value of alignment above the mean (0.93) were considered misaligned, with respect to the mean.

We further sought to categorize our sample of fifteen classes into two groups, those that were more aligned and those that were less aligned. To create these group, we identified a natural break in the distribution of alignment scores, which roughly corresponded to the mean alignment score, 0.93. We classified class sections with alignment score up to 0.93 as above average alignment, or what we called “aligned,” while classes beyond the mean alignment score (> 0.93) were below the average, and we called “misaligned” (with respect to our sample mean; Fig 1). The LO and assessment Bloom scores and alignment scores represent three of the four independent variables in our analysis.

### Learner-centeredness of classroom instruction

During Fall of 2013 and Spring of 2014, we recorded 86 classroom sessions, representing 6–10 recordings of each instructor. Most filming days were randomly selected and most instructors were not notified in advance. Of the useable, full length recordings, we randomly selected three or four videos from each section to evaluate using the Reformed Teaching Observation Protocol (RTOP) [62]. We chose to evaluate several classes within a section to better capture the range of pedagogies each instructor may use through a semester; thus, representing the actual learner-centeredness a student from that class may experience. RTOP is a validated [62, 63] and reliable [64–65] quantitative method to evaluate the learner-centeredness of instruction. Eight raters were trained on RTOP by a certified, external RTOP trainer. Each video was independently rated by at least two raters and the inter-rater reliability was high (Generalizability Coefficient = 0.787; according to Putka et al. [66]), which was comparable to the training intra-class correlation coefficient of 0.911. Any numerical difference in reliability coefficients is merely a reflection of the difference in research design (i.e., fully crossed in the training data and neither fully crossed nor nested in the test data) not a reduction in rater agreement following the training.

RTOP scores for each section were averaged into a single score. The average score for each section was categorized into one of five RTOP levels: I (raw score = 15–30; strictly teacher-centered lecture); II (31–45: teacher-centered lecture shifting toward learner-centered classroom with student involvement); III (46–60: learner-centered classroom); IV (61–75; learner-centered with critique); and V (76–100; complete learner-centeredness involves critique, open-ended inquiry, hypotheses, and critical reflection) [42, 67]. We used raw RTOP values as a continuous explanatory variable in regression analyses and RTOP level as a categorical explanatory variable in ANCOVA analyses.

### Data adjustments and analysis

We excluded three questions of the cognitive skills quiz for our final analyses. These exclusions were made because of slight wording differences between the pre and post-quizzes that created ambiguity in a single best response (chromosome question); the Bloom level was too low (climate change question); and students performed poorly, average score being 10% correct, and their performance worsened through time (cheetah/salamander question) (Table 1). From the remaining nine questions, the highest possible score was nine points. Of the 1114 student participants, 620 students completed both pre and post-quizzes. Although non-response bias can be problematic in studies with volunteer participation, our response rate of 55.6% is very close to the accepted average response rate in psychological studies [68]. We conducted our analysis using only 313 of the paired student responses, because the remaining 307 paired students’ responses included ambiguous responses for one or more of the free-response questions (ginseng/iron and/or spider monkey/Grandma Johnson). For example, answers such as “N/A”, “I don’t know”, and nonsensical text were coded as missing data rather than zeros; thus, not included in our analysis. We feel this data reduction was necessary to maintain the quality of the data. The mean pre-quiz scores of all 620 pairs compared to the reduced 313 pairs was merely 0.26 points lower. This reduction is expected because all ambiguous free-responses are coded as zero in the full 620 pairs, which lowers the average. Our reduction to the 313 pairs with complete responses, therefore, is likely a good representation of the larger sampled population.

We sought to avoid problems associated with pseudo-replication, so our unit of analysis was class section not individual student [69]. Therefore, among our 313 total students, the fifteen sections had on average 19.9 sampled students (minimum = 5 sampled students per section, maximum = 99 per section). Within each section, we averaged the individual student pre-quiz scores and then averaged post-quiz scores to represent two responses for each section. We believe this aggregation was appropriate, and clustering effects within sections was negligible, because the variance in pre-quiz scores among sections is nearly identical to the variance within sections (F_14,298_ = 0.98, *p* = 0.48). We used an ordinary least squares regression and ANCOVA in SAS 9.3; these techniques were appropriate because our data met normality requirements (Shapiro-Wilks, W = 0.96, *p* = 0.66), and a plot of standardized residuals versus predicted values indicated that the assumption of equal variances was reasonable. Pre-quiz scores served as the covariate and learning objective Bloom scores, exam Bloom scores, and RTOP scores or level were the independent variables explaining the post-quiz scores. A Tukey-Kramer post hoc analysis helped determine which groups differed. We also ran outlier analysis and sensitivity analysis to evaluate suspected outliers. For our final analyses, we opted to retain the one outlying section because the magnitude of response was similar when it was removed or retained. Moreover, this section is only an outlier because the student scores fall well below the average response, yet it maintains the positive correlation between pre and post-quiz scores, and there was no reason to restrict the range of possible scores.

## Results

### Participating students and class sections

Of the 313 students in our final analysis that submitted complete demographic data on pre and post-quizzes, 41.9% (131 students) were freshman, 38% (119) were sophomores, 13.7% (43) were juniors, 5.1% (16) were seniors, and 1.3% (4) were post baccalaureate. Of those that reported their grade-point average, the mean was 3.4 on a 0.0–4.0 scale, while the mean self-reported ACT score was 23. Our student population was 86% Caucasian, 6% Latina/o, and 8% other ethnicities or unreported. On average, these students had taken one previous biology class in high school, and 20% had previously taken a college biology course.

On average, 26.1% of students in each section participated and were used in the final analyses. The results presented from here forward reflect means of students within a section, as class section was our unit of analysis. Across all sections, the mean score on the pre-quiz was 1.7 out of 9 questions (19.2% accuracy). On a question-by-question basis, the level of accuracy on the forced response questions was less than random guessing, indicating purposeful selection of incorrect responses. The mean post-quiz score for the fifteen sections was 2.6 out of 9 questions (29.2%). The mean difference in quiz scores, 0.9 points or a 52% improvement, was significantly different than zero (t_14_ = 7.65, *p* < 0.001).

We found the mean learning objective Bloom score among the nine faculty teaching our fifteen sections was 2.5 (on a scale of one to six) with scores ranging from 1.6 to 4.7. The mean assessment Bloom score by instructor was 2.0 with scores ranging from 1.5 to 3.8. Of our fifteen sections, we classified ten classrooms as aligned (above the mean) and five as misaligned (below the mean), with the mean alignment score being 0.93 (Fig 1). This mean alignment score includes the redundancies inherent in six of our participating instructors teaching more than one section (the mean alignment score of the nine instructors without duplicates was 0.99). While the possible range of alignment scores was zero (perfectly aligned) to five (completely misaligned), alignment scores in our sample ranged from 0.12 to 2.95. The learner-centeredness was evaluated for all fifteen class sections, and the mean RTOP scores ranged from 30.2 (RTOP category I) to 54.4 (RTOP category III) with an average mean RTOP score of 38.9 (category II). RTOP scores were linearly correlated with assessment Bloom scores (Pearson *r* = 0.63) and the number of students in each section that participated in our study (Pearson *r* = 0.64).

### Bloom scores and alignment

**Fig 2:**
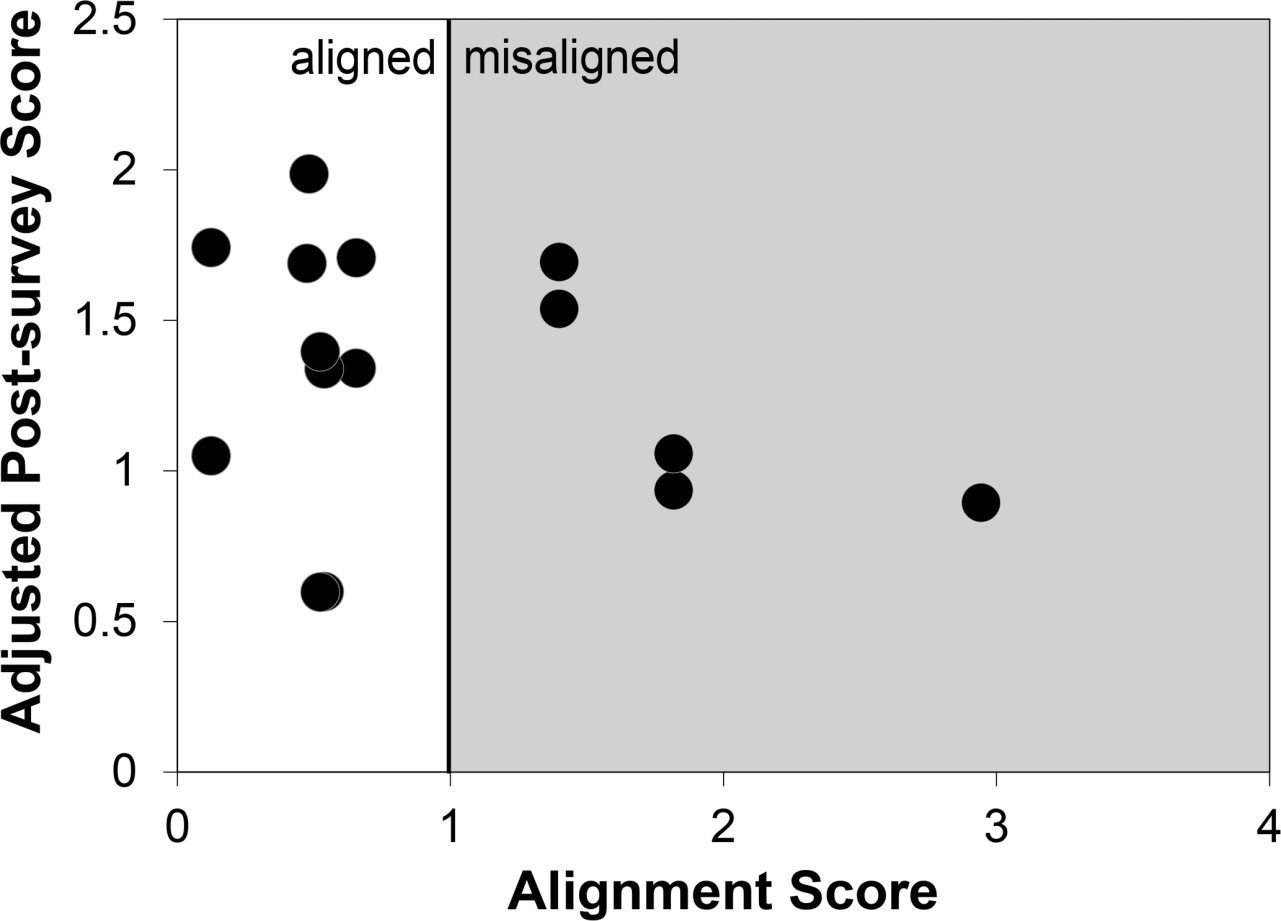
Scatterplot of alignment scores and adjusted post-quiz scores. Little correlation (*r* = -0.28) exists between alignment and students’ post-quiz scores, that are adjusted for pre-scores.

Bloom scores did not explain variation in students’ post-quiz scores by section (learning objective Bloom scores: F_1,9_ = 1.13, *p* = 0.31; assessment Bloom scores: F_1,9_ = 0.83, *p* = 0.38). Alignment and Bloom scores could not be included in the same model due to severe multicollinearity (e.g., Pearson *r* = 0.94 between alignment and learning objective Bloom scores), because Bloom scores were the basis of alignment calculations. All ANCOVA models including alignment suggested that this variable did not play a significant role in improving students’ post-quiz scores (F_1,10_ = 0.84, *p* = 0.38). Additionally, no interactions were found between alignment and RTOP scores (F_1,9_ = 0.01, *p* = 0.92). Similarly in our regression analysis, we found that alignment did not explain variation in students’ post-quiz scores (F_1,11_ = 2.69, *p* = 0.13; Fig 2), nor did it interact with RTOP scores (F_1,8_ = 0.21, *p* = 0.66). Thus, assessment Bloom scores, learning objective Bloom scores, and alignment were removed from our final models.

### Pre-quizzes and RTOP

**Fig 3:**
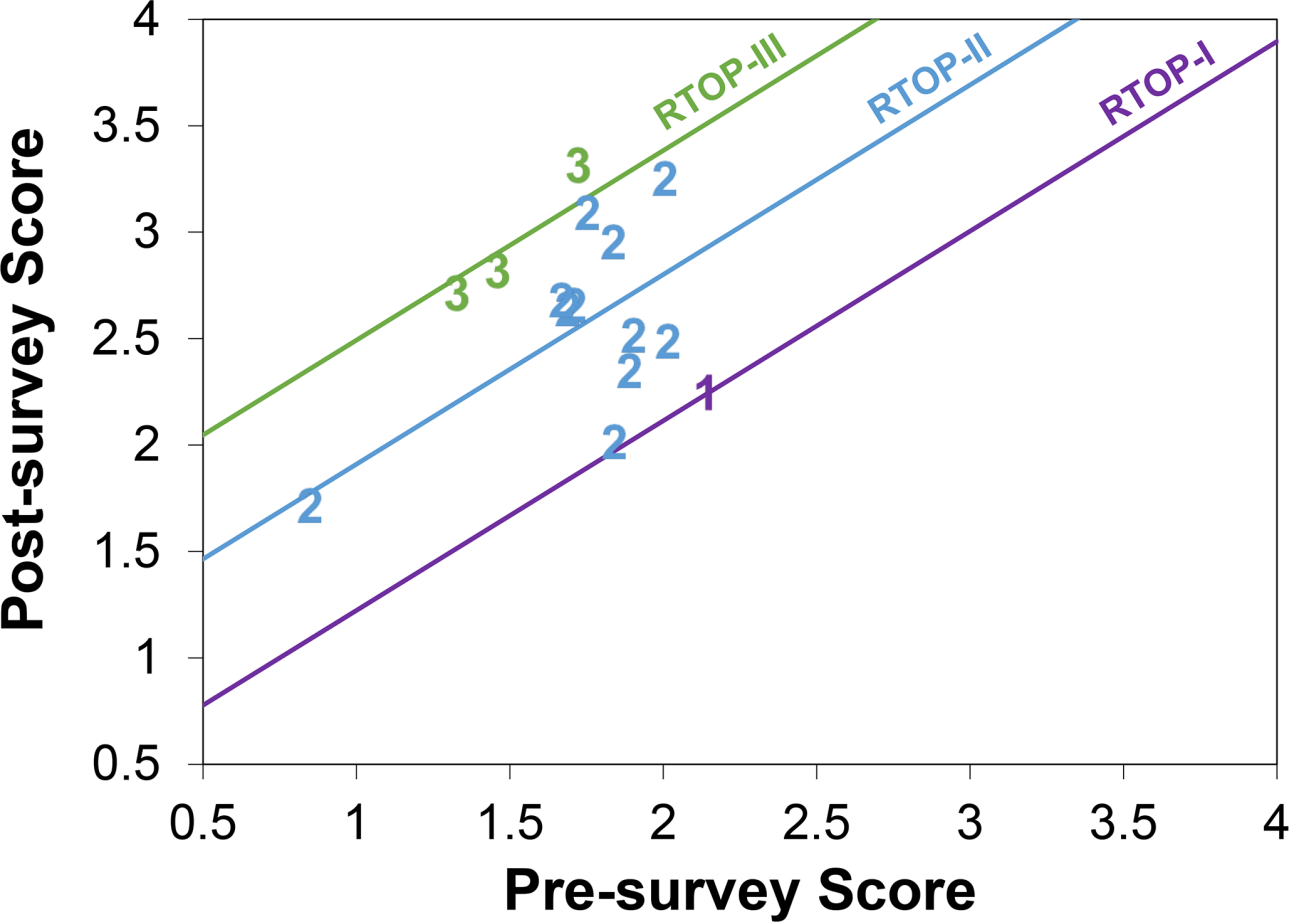
Scatterplot of pre versus post-quiz scores, with three RTOP regression lines. Purple line and “1” symbol represents classrooms classified as RTOP level I (straight lecture). Blue line and “2” symbols represent classrooms classified as RTOP level II (lecture with some demonstration and minor student participation). Green line and “3” symbols indicate classrooms classified as RTOP level III (students actively participating in activity and critique of information).

The final ANCOVA model to best explain variation in student post-quiz scores, by section, included pre-quiz scores as a covariate (F_1, 11_ = 7.17, *p* = 0.02) and RTOP level (F_2, 11_ = 4.53, *p* = 0.04; Fig 3). We found that the slopes of each RTOP category were not significantly different (*p* = 0.68), thus our model assumed the slopes for each of the three RTOP levels were equal (Fig 3). From a *post hoc* Tukey-Kramer adjustment for multiple comparisons test, we concluded that RTOP level III was better than both the RTOP I (t_11_ = -2.79, *p* = 0.04) and RTOP II categories (t_11_ = -2.42, *p* = 0.08) in explaining post-quiz scores. However, RTOP levels I and II were not statistically different (t_11_ = -1.77, *p* = 0.22).

In our final regression model, RTOP (F_1,12_ = 4.85, *p* = 0.04) and the pre-quiz scores (F_1,12_ = 5.34, *p* = 0.05) both explained a significant amount of variation in student post-quiz scores (R^2^ = 0.38). Using this relationship, a 7-point increase in RTOP scores, which corresponds to roughly a half of an RTOP level, would result in a 0.22 point average increase on our 9-item quiz (or an 9% average increase) in post-quiz scores, while a 14-point increase in RTOP score, a full RTOP level, would result in a post-quiz score average increase of 0.44 (or 19%).

## Discussion

### Education works

On average, the sampled students’ scores from our fifteen sections increased by over 50% from pre to post-quizzes. While their overall mastery of biology higher-order cognitive skills, as measured by our instrument, appeared poor (i.e., final mean score had less than 30% accuracy), their performance was comparable to, or only slightly lower than, students sampled in the original concept inventories we used [52, 56–59]. Any discrepancy in performance of our student population is likely due to differences in course level and intensity. Specifically, the classes we sampled were introductory non-majors biology that briefly covered all major topics from molecules to ecology, while some of the work validating the concept inventories used specialty classes (e.g., Genetics, Cell and Molecular Biology), which allowed those students to invest more time on the material.

Further, we believe the apparent difficulty of our instrument was beneficial to capture small scale difference and avoid the ceiling effect (i.e., the highest individual student post score was 7.1 out of nine) [70]. Our cognitive skills quiz was a proxy for skills improvement, thus the relative improvement, and not the raw scores, was the best measure of gains in students’ high-order cognitive skills. Additionally, we found no relationship between student performance and class time, enrollment, or the content area strength of each faculty member. Our data support our first hypothesis that across sections, students’ higher-order cognitive skills improve following a semester of biology instruction, regardless of the type of instruction.

### Assessments are not the clear answer

An increase in students' higher-order cognitive skills, as defined by performance on our quizzes, was not associated with an increase in the cognitive level of assessments. This finding was surprising because it challenges previous work [24–25, 71] that suggest an integration of higher-level assessments can promote critical thinking. This contradiction may be due to four possible factors or a combination thereof. First, our study measured only high-stakes summative assessments. Summative assessments provide a mechanism for students to demonstrate their proficiency of the skills they have honed throughout their learning experience, but provide little feedback for improvement [72]. Yet higher-order cognitive skills must be practiced and developed over time, and it is unclear if our sample of classes offered consistent feedback through formative assessments as a mechanism to improve these skills.

Second, the vast majority of our instructors administered assessments that were, on average, at low Bloom levels (Fig 1). While this preponderance of low-level assessments has been documented elsewhere [28–29], the signal for this factor in our search for the best models may have been weakened by not having all possible Bloom’s levels. Jensen et al. [26] found that students trained with high-level assessments through the semester performed better on both high and low-level assessments beyond peers only trained at low levels. Their study suggests that future quasi-experimental studies integrating a more complete range of Bloom level assessments in combination with a range of learner-centered to teacher-centered classroom environments may better clarify any potential role of assessments on student critical thinking, beyond the gain from in-class pedagogies.

Third, most of the assessment items analyzed in our study were multiple-choice questions. Palmer and Devitt [73] suggest that carefully crafted multiple-choice questions can replace essay questions to test higher-order skills. Yet multiple studies oppose this view, and find that development of students’ higher-order cognitive skills is hindered by the use of multiple-choice exams [74–75]. Our sampling of classes, where all but two instructors used strictly multiple-choice assessments, is likely representative of introductory science courses. In 2011, a national survey suggested that 29.3% of college instructors strictly use multiple choice exams [76], and the percentage invariably increases in large enrollment, introductory science courses [75]. Perhaps the format rather than the cognitive level of assessments is more important in maximizing student learning. Multiple-choice assessments tend to capture memory learning; whereas, performance assessments may provide a better measure of critical thinking [77]. For example, arguments-from-evidence has been associated with higher levels of sophistication in understanding [78], and concept maps have been shown to assess both declarative and procedural knowledge [79]. The success of these varied formats, and their focus on performance, prompts further investigation into the role of assessment.

Fourth, while we found assessments do not explain variance in students’ post-quiz scores of higher-order cognitive skills, our rating of assessments were based solely on the cognitive process dimension of Bloom’s learning taxonomy [10]. Future work that also integrates an analysis of the knowledge dimension (i.e., factual knowledge, conceptual knowledge, procedural knowledge, or meta-cognitive knowledge) [10] may lend additional insight into how assessments, and what format of assessments, contribute to building students’ higher-order cognitive skills [38].

### Alignment at this level may not matter

Although learner-centered teaching dogma asserts that alignment promotes student learning [36–40], we found no direct link (Fig 2). Literature on test expectancy theory presents a similar conundrum; whereby, research both supports and refutes congruency in students’ expectation and their performance on certain types of exams [80–81]. However previous test expectancy research primarily focuses on the form of assessment (i.e., essay versus multiple-choice) and not the cognitive level of those assessments [81]. The only other study that has compared student performance against the cognitive level of assessments, held the level of learning objectives and in-class pedagogies constant [26]; thus, only one of their treatment conditions was fully aligned (i.e., high level learning objectives, inquiry-based in class practices, and high level assessments). While students in Jensen et al.’s [26] fully aligned group outperformed students in the misaligned group on both low and high-level portions of a final exam, the relative contributions of alignment versus the testing effect of weekly high-level exams and multiple high-level unit exams is unclear.

While we found learning objective Bloom score was also not a good predictor of student critical thinking, it was likely not the sole reason why alignment had low explanatory power. The majority of our classrooms were aligned at low levels (i.e., mean Bloom score for assessments was at the Knowledge level and mean learning objective score was between the Knowledge and Application levels). Rather, we suspect that the interaction of alignment and the cognitive level of assessments or learning objectives represents a better window into how to improve students’ critical thinking than the main effect of alignment. Specifically, alignment at high cognitive levels may increase student learning by signaling which skills and knowledge are expected of students, and preparing them for these expectations through modeling and practice. These signals are especially important because students tend to have low cognitive level expectations in biology education [82]. So alignment at low cognitive levels, which was exemplified in most of our participating classes, likely does not benefit student learning because students do not expect to be challenged and then they never are. Whereas classes aligned at high cognitive levels, provides clearly defined expectations and follows through with assessments that actually test students at that level. Although our study lacked well-aligned classes at high Bloom levels, we predict future studies which incorporate this treatment combination would find students in aligned classrooms at high levels may demonstrate more of a change in higher-order cognitive skills than all other treatment combinations. Clearly, alignment is a good tool for instructors to formulate their course materials to ensure students reach the desired outcomes [33]; however, its value in student learning may only be focused at higher cognitive levels. Additional research, which combines all levels of alignment and the cognitive level of assessments and learning objectives, is needed to test this hypothesis.

### Student-centered teaching trumps all

Improvement of higher-order cognitive skills requires time, practice, and guidance. In a teacher-centered classroom (i.e., RTOP level I or II), the majority of class time is dedicated to lecture, and information is disseminated uni-directionally. If higher-order cognitive development is expected, students must develop these skills outside of class without feedback or modeling from their peers or instructor. Whereas in a student-centered classroom (i.e., RTOP level III or above), ample time is provided for students to work in small groups testing hypothesis, defending arguments, and formulating solutions to problems.

We found a clear link between more learner-centered in-class pedagogies and improvements in students’ critical thinking (Fig 3). This finding, however, is not new. Previous work in the science, education, and psychology literature has resoundingly demonstrated that more active, learner-centered approaches can improve student performance [11–16, 18], reduce failure rates [11], improve metacognition and motivation [83–84], and promote critical thinking [44, 85]. While our study further contributes to this growing body of evidence supporting the efficacy of learner-centered teaching, no other study has weighed the relative effect of this type of in-class instruction against other pedagogical elements. Specifically, previous work has established that active, learner-centered approaches are important; however, our findings identify in-class practices as the main driver leading to improvements in students’ higher-order cognitive skills, above classroom alignment and above the cognitive level of assessments and learning objectives.

Our findings indicate that reforming a classroom by one RTOP level is associated with a nearly 20% improvement on students’ higher-order cognitive skills. A notable limitation of our work is that the majority of our participating instructors fell into RTOP level II, and only a few were classified as RTOP III and none were in RTOP IV or V. While our work suggests a fairly even student gain with each increasing RTOP level (Fig 3), we have no data to understand the shape of this relationship beyond an RTOP level III. Future study, including these highly reformed classrooms, could model if student critical thinking gains continue linearly with RTOP levels above III or if perhaps there is a plateau, at which point there are diminishing student returns with increasing classroom reform.

### Recommendations

Faculty development is a critical component for institutional change and fostering improved student outcomes [42, 86, 87, 88]. The focus of such training may include many components of pedagogy that we investigated in this study, including alignment and the design and implementation of learning objectives, assessments, and in-class activities. We may have observed higher student outcomes in the classes we sampled if there had been a greater emphasis on formative assessments with regular, individualized feedback [89]. For faculty, formative assessments may act as a bridge between learning objectives and in-class activities; where feedback signals which instructional activities are appropriate to meet the desired expectations. For students, formative assessments may act as a bridge between in-class activities and summative assessments; where feedback guides study behaviors to center on weak areas. Formative assessment may also scaffold the “transition from teacher-supplied feedback to learner self-monitoring” [61], which can promote critical thinking in the long-term. Clearly, the inattention to formative assessments in many of our studied classrooms was a flaw, yet not uncommon in many science classrooms. Further, many other learner-centered strategies beyond assessment can be the focus of successful faculty development.

Given time constraints and the effort required to keep current on discipline-based education research, it is often unreasonable to assume an instructor can improve in all areas of teaching at once. Instead instructors will likely weigh which elements of learner-centered pedagogies they will reform first. Our study indicates that in these scenarios, in-class student-centered teaching will positively impact students far more than any other component of pedagogy. While this finding is novel and informative, we caution readers to view our data and interpretations with caution due to necessary data adjustments outlined previously.

Although we found no evidence that the degree of alignment nor the cognitive level of assessments explained significant variance in students’ higher-order cognitive skills, each has an important role in teaching and learning. We propose two potential roles. First, inclusion of both high and low Bloom level items on assessments may aid instructors in identifying those skills with which their students need the most help. Inevitably students receive less instruction, thus less structured practiced, with higher level thinking. Therefore constructing assessments heavy with high Bloom level items and appropriate feedback may provide this needed exposure. Previous work suggests that regular testing with primarily high Bloom level items allows students to excel at both lower and higher-order cognitive skills [26, 52]. The use of formative assessments with high Bloom level questions may enable students to become metacognitive about their own strengths and weaknesses, while there is still time to improve [22, 24].

Further, it is important to untangle the misconception that Bloom cognitive levels are synonymous with a scale of difficulty. Lemons and Lemons [90] attest that question difficulty is not positively correlated with the cognitive level of the item. Then, by extension, development of higher-order cognitive skills may be relevant for both high and low achieving students, depending on the instructor’s goals [74]. Regardless, higher-order questions should not be avoided simply because the course is introductory. Their notable absence, especially in introductory science classes, may also arise because instructors often struggle to craft successful higher-order assessment questions [90, 91]. Therefore, efforts to train faculty in these skills may both increase instructor’s expectations and improve student learning.

Second, we believe that alignment may improve student learning indirectly by helping instructors plan curricula. Momsen et al. [43] document that faculty tend to write learning objectives at high Bloom levels. Through backward design [40], alignment at high cognitive levels may encourage faculty to craft assessments to a level to match their expectations, rather than administering exams fraught with low cognitive level questions which seem to be the norm [29]. Accordingly, instructors may incorporate more learner-centered pedagogies in class to align with high-level objectives and assessments, which then allows students to practice these skills, and, as we have found, promote critical thinking.

In summary, we suggest that the most effective strategies to promote student critical thinking include alignment at a high Bloom level using learner-centered in-class activities and well-balanced learning objectives and assessments, including high Bloom level items. Active in-class pedagogies resounding lead to a successful classroom environment, and future research will help clarify the contributions of assessments and alignment to the development of student’s higher-order cognitive skills. Additional study with other student populations and a similar, non-observational study which controls each factor step-wise is needed to validate our findings.
